# Exploring the potential of contrast agents in breast cancer echography: current state and future directions

**DOI:** 10.1007/s40477-023-00809-0

**Published:** 2023-08-11

**Authors:** Oriana Monzeglio, Vittoria Maria Melissa, Sara Rodolfi, Eleonora Valentini, Alessandro Carriero

**Affiliations:** 1 Department of Diagnosis and Treatment Services, Radiodiagnostics and Interventional Radiology, AOU Maggiore Della Carità, Corso Mazzini 18, 28100 Novara, Italy; 2 Department of Translation Medicine, University of Eastern Piemonte UPO, Via Solaroli 17, 28100 Novara, Italy

**Keywords:** Breast cancer, Neoangiogenesis, Ultrasound contrast agents, Microbubbles

## Abstract

Breast cancer stands as the most frequent malignancy and leading cause of death among women. Early and accurate detection of this pathology represents a crucial factor in enhancing both incidence and mortality rates. Ultrasound (US) examination has been extensively adopted in clinical practice due to its non-invasiveness, affordability, ease of implementation, and wide accessibility, thus representing a valuable first-line diagnostic tool for the study of the mammary gland. In this scenario, recent developments in nanomedicine are paving the way for new interpretations and applications of US diagnostics, which are becoming increasingly personalized based on the molecular phenotype of each tumor, allowing for more precise and accurate evaluations. This review highlights the current state-of-the-art of US diagnosis of breast cancer, as well as the recent advancements related to the application of US contrast agents to the field of molecular diagnostics, still under preclinical study.

## Introduction

Breast carcinoma is the most frequent malignant neoplasm in women, accounting for 25% of all cancers and representing the leading cause of cancer-related mortality, responsible for 14.3% of cancer deaths [1]. In 2018, the International Agency for Research on Cancer estimated that over 2 million new cases of breast cancer were diagnosed globally [2].

The incidence of breast cancer has increased significantly over the past century, possibly due to changes in lifestyle, social and cultural environment, and early detection [2]. In Italy, the incidence trend has slightly increased by 0.3% per year, while mortality rates have continued to decline. According to EUROCARE 5, the 5-year survival rate for the period 2005–2009 was 87%, higher than the European average of 82% for diagnoses between 2000 and 2007 [3].

The prognosis and survival rates of breast cancer vary considerably based on the stage of the tumor, histopathological type, and molecular phenotype. The effectiveness of existing treatments largely depends on these factors, highlighting the importance of accurate early diagnosis. Furthermore, prevention measures, such as screening programs and improving therapies, are essential in increasing survival rates [4]. It is therefore crucial to be able to carry out a correct and accurate early diagnosis.

## Tumour neoangiogenesis

The survival and growth of tumours require nutrients and oxygen supplied by nearby capillaries. Since the oxygen diffusion limit is only 100–200 μm, rapidly expanding tumors must recruit new blood vessels to grow beyond the critical threshold, thus avoiding hypoxia and acidosis that would otherwise lead to cell death through apoptosis and necrosis [5–7]. This process is known as neoangiogenesis, which is largely conducted by endothelial cells (ECs) and triggered by tumour cells releasing angiogenic factors [6, 7].

During the early 1960s, researchers proposed the first hypotheses suggesting that tumors had the capacity to produce certain substances that could diffuse into the interstitium, laying the foundation for further research in this field. Several years later, in 1971, Folkman demonstrated that tumour growth and metastasis development were angiogenesis-dependent processes [8]. Now it is well-established that, under physiological conditions, there is a balance between pro-angiogenic and anti-angiogenic molecules. However, when the "angiogenic switch" occurs, there is a prevalence of pro-angiogenic factors [6]. Once the tumour has established its angiogenic state it grows much more rapidly and is more prone to developing metastases [7].

**Fig. 1:**
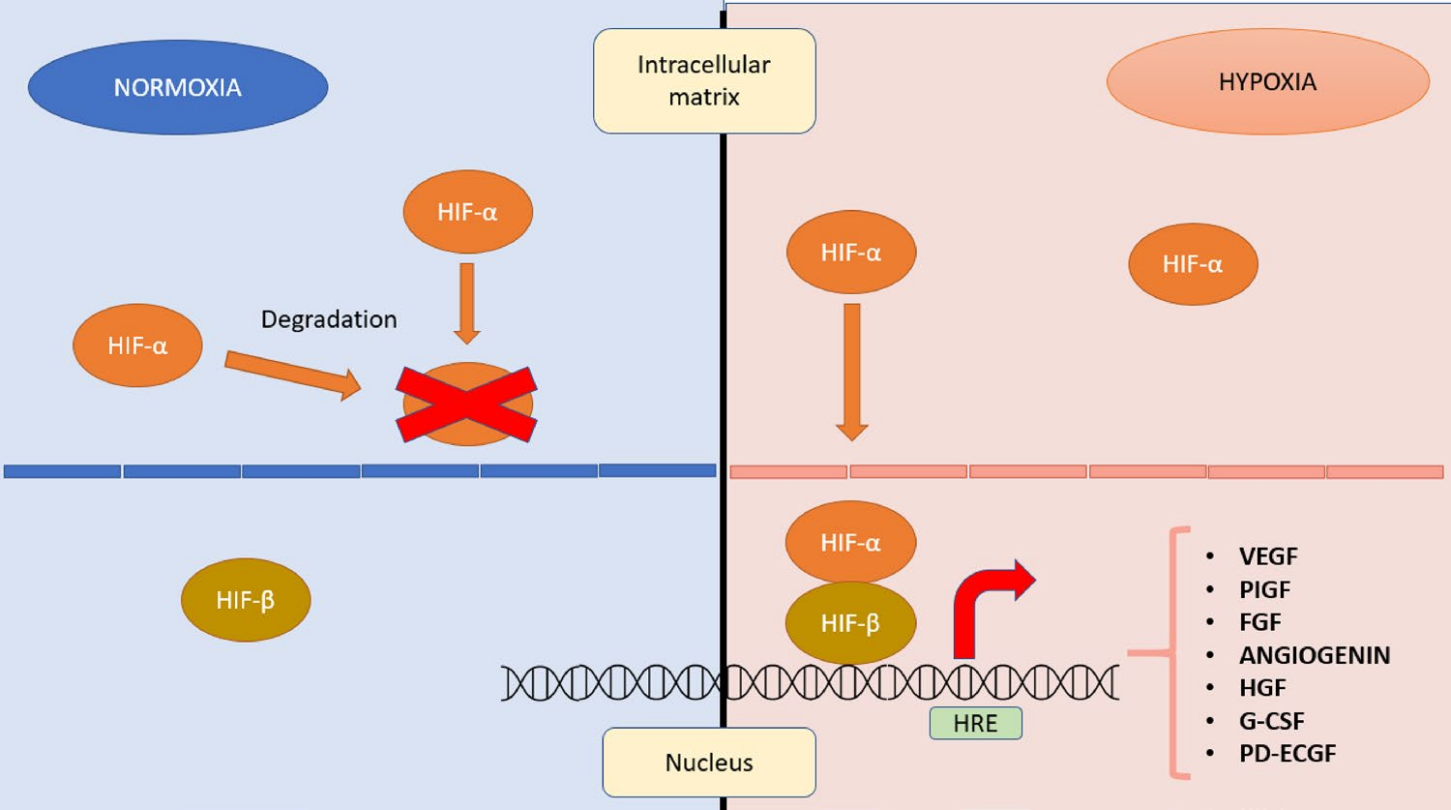
(left) Under normoxic conditions, the 1α subunit is produced and degraded in the intracellular environment. (right) Under hypoxic conditions, the degradation of HIF-1α is blocked, allowing its accumulation and translocation into the nucleus where it associates with the 1β subunit to become an active transcription factor

There are several stimuli that favor neoangiogenesis, but hypoxia is probably the most important one [6, 7]. Hypoxic tumour cells express high levels of specific transcription factors, including hypoxia-inducible factor (HIF), consisting of two subunits: 1β, which is constitutively expressed, and 1α, which is produced and rapidly degraded under normoxic conditions. Under hypoxic conditions, on the other hand, the degradation of HIF-1α is blocked, allowing its accumulation and translocation into the nucleus, where it binds to the 1β subunit. Together, the two subunits constitute the active transcription factor, capable of promoting the transcription and release of various pro-angiogenic growth factors such as vascular endothelial growth factor (VEGF), placenta growth factor (PIGF), fibroblast growth factor (FGF), angiogenin, interleukin-8, hepatocyte growth factor (HGF), granulocyte colony-stimulating factor (G-CSF), and platelet-derived endothelial cell growth factor (PD-ECGF), as shown in Fig. 1 [6, 7, 9].

Among the different growth factors activated, VEGF has been recognized as one of the most effective. In fact, it is more correct to refer to the VEGF gene family, which includes six different glycoprotein homodimers [5, 7, 10].

The first gene of the family to be identified was VEGF-A, which is also the most involved in angiogenic activities in mammalian cells. This gene gives rise to different mature protein isoforms of VEGF-A through alternative splicing from a single mRNA precursor, which differ from each other in amino acid length. The isoform of 165 amino acids is the most active and abundant one as it is expressed by a large variety of normal and tumour cells, including breast cancer cells. Moreover, the expression of the VEGF-A gene can also be regulated by hypoxia as the gene contains a hypoxia responsive elements (HRE). Hypoxia is therefore able to induce a rapid increase in mRNA levels of the gene [10, 11].

To date, three types of receptors for VEGF have been described. In all cases, they are transmembrane receptors belonging to the tyrosine kinase family. VEGFR-2, also known as KDR (kinase-insert domain-receptor), is the variant mainly expressed by ECs and is responsible for most of the effects induced by VEGF-A on these cells, including microvascular permeability, proliferation, migration, invasion, and survival [11].

**Fig. 2:**
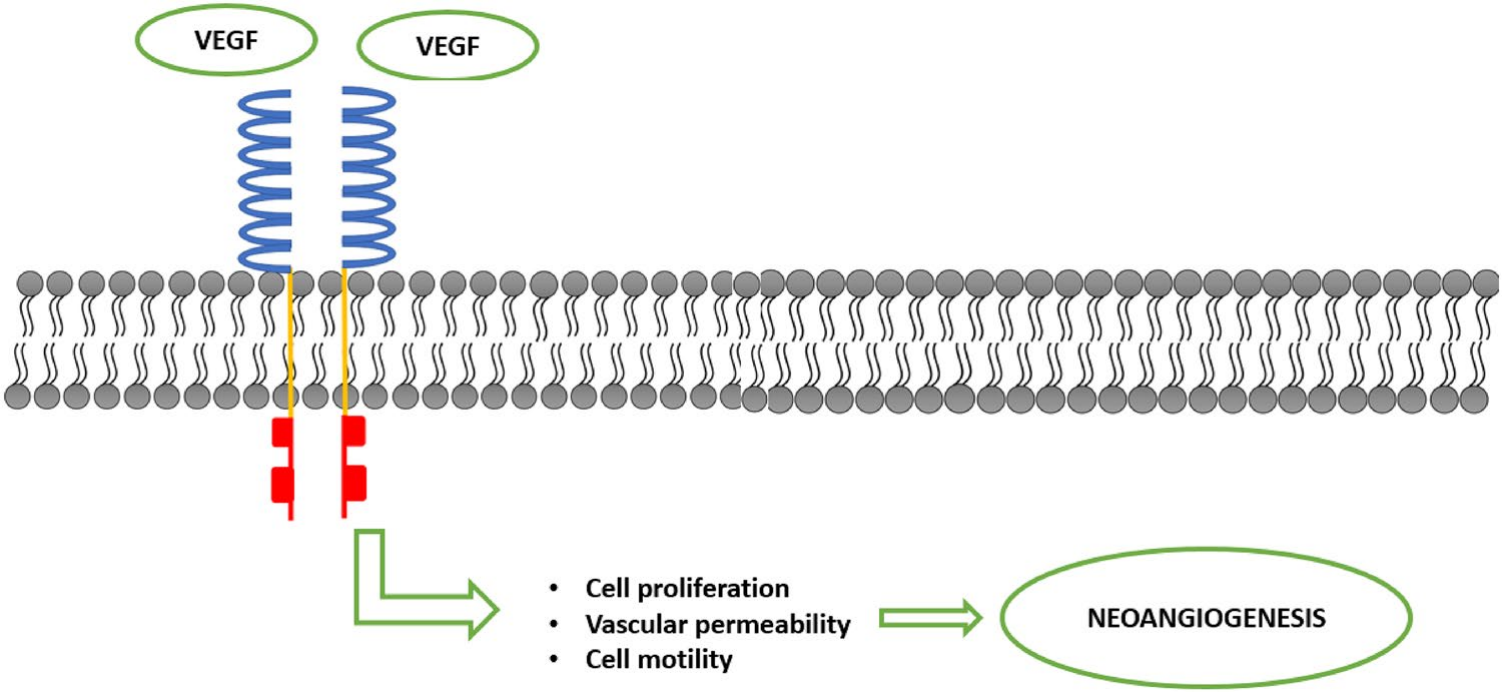
VEGFR2 is a tyrosine kinase receptor composed of a small transmembrane region, an intracellular portion, and an extracellular portion with seven immunoglobulin-like domains. Activation of the receptor stimulates the proliferation and motility of ECs, leading to the formation of new blood vessels and increased vascular permeability

Like all tyrosine kinase-type growth factor receptors, VEGFR2 is a receptor consisting of an extracellular portion with seven immunoglobulin-like domains, a small transmembrane portion, and an intracellular portion containing the tyrosine-kinase domain (Fig. 2). The interaction with its ligand induces the dimerization of the receptor itself, allowing the autophosphorylation of specific tyrosine residues on the intracellular side and the activation of downstream signaling pathways triggering all the biological effects described above [10].Dysregulated tumour angiogenic process differs significantly from normal angiogenesis, which requires a delicate balance between pro-angiogenic and anti-angiogenic factors to facilitate the formation and maturation of new vessels. During tumour neoangiogenesis, the loss of this balance results in the rapid proliferation of ECs and the formation of structurally and functionally altered vessels. These newly formed vessels are disorganised and irregular, characterised by tortuosity, dilation, fenestrations, and arterio-venous shunts that lead to abnormal and inefficient perfusion. Additionally, the lack of sphincters and pericytes renders these vessels largely independent of the normal mechanisms regulating capillary blood flow. The basement membrane is either discontinuous or absent altogether, further increasing the permeability of tumour vessels, particularly to macromolecules. The degree of disorganisation of tumour vessels and the rate of tumour growth are directly proportional, with greater randomness observed in more rapidly growing tumours [5–7].

## Breast ultrasound

Breast ultrasound (US) is a commonly used imaging technique for diagnosing breast cancer due to its safety, non-invasiveness, low cost, and absence of ionizing radiation. The use of US in breast imaging began in the late 1970s with the B-mode technique [12]. Since 2003, with the addition of a specific chapter on guidelines for acquiring US images and interpreting them in the Breast Imaging Reporting and Data System, the use of US has increased significantly [13].

US examination can differentiate benign masses from malignant lesions by analysing the main anatomical characteristics, such as morphology, orientation, internal structure, and lesion margins, which can be investigated in multiple planes with high resolution in both adipose and dense breasts with a marked glandular component [13, 14]. However, architectural features alone do not provide enough information about the nature of the tumour, such as its potential for invasiveness, growth, and metastasis. Functional alterations, which occur before the morphological ones become visible to diagnostic imaging, are crucial for early diagnosis, as in the case of neoangiogenesis (Table 1). Therefore, relying exclusively on anatomical features is insufficient for accurate early diagnosis [5, 15, 16].

**Table 1:** Primary ultasound criteria for breast cancer diagnosis [17]

Lesion	Malignant	Benign
Shape	Irregular	Ovoid, round
Orientation	Vertical, taller than wider, indifferent	Parallel, wider than taller
Margins	Indistinct	Circumscribed, well defined, thin, echogenic capsule,
Contours	Irregular, spiculated, angled	Smooth, three or less lobulations
Echogenicity	Markedly hypoechoic	Hyperechoic, isoechoic, or moderately hypoechoic
Calcifications	Microcalcifications	Absent
Surrounding tissue	Distorsions	Compression, no distorsions
Retraction	Present	Absent

Recently, additional techniques have been developed to increase the diagnostic power of B-mode US by studying the functional alterations of tumours, including neovascularisation [5, 12, 13].

## Echo-colour-Doppler

The utility of echo-colour-Doppler evaluation in breast imaging is currently a matter of debate.

In the early 1990s, echo-colour-Doppler evaluation was introduced as an adjunct to B-mode US assessment, aiming to differentiate between benign and malignant pathology based on the presence of intralesional vascularization and its morphology.

Intralesional vascularization is assessed by the presence of intralesional echo-colour-Doppler signals. Initial studies showed that nearly all malignant masses were hypervascularised and contained echo-colour-Doppler signals, while only a small percentage of benign lesions exhibited this feature [18]. However, advances in technology have later demonstrated that echo-colour-Doppler signals can also be detected in benign lesions [19].

Another important characteristic of echo-colour-Doppler evaluation is the study of the distribution of vessels within the lesion. Studies from the late 1990s put forward that the presence of penetrating vessels suggested malignancy, while peripheral vascularisation indicated benign lesions [20]. Nevertheless, subsequent studies raised questions about the accuracy of this characteristic, leading to the removal of echo-colour-Doppler evaluation from the ACR BIRADS guidelines in 2013 [1].

Recently, Watanabe and co-workers have addressed the controversial issue of the clinical relevance of echo-colour-Doppler, showing that echo-colour-Doppler vascularisation signals are clinically significant when considered in the context of the patient's age. Specifically, masses with low vascularisation tend to be benign, regardless of age. In contrast, masses with high vascularisation are more likely to be malignant in patients over 50 years of age, while in patients under 50 years of age with the same degree of vascularisation the likelihood of malignancy decreases. Thus, interpreting echo-colour-Doppler results in conjunction with the patient's age increases the US specificity, underscoring the effectiveness of echo-colour-Doppler in the diagnosis of breast cancer [19].

### CEUS

Contrast-enhanced US (CEUS) is a potential diagnostic tool for malignant breast tumours due to their generally high vascularisation. This method provides real-time information on intralesional vascularisation and blood flow by injecting intravenously a contrast medium consisting of microbubbles [5]. These microbubbles are composed of inert gases (*i.e.,* primarily fluorinated gases) that have a longer dissolution time in the blood due to their lower solubility in ambient air, resulting in greater stability and a longer half-life in vivo. The microbubbles are stabilised by an outer phospholipid shell and have a size range of 1–5 microns, which makes them exclusively intravascularly localised and useful for detecting pathological processes involving cells present in this site, such as ECs [21]. When the microbubbles are insonated, they undergo non-linear compression and rarefaction movements, resulting in a harmonic signal that is distinguishable from the background signal and displayed as a contrast-agent only image. Following injection, the microbubbles have a relatively short half-life ranging from a few minutes to 30 min, depending on the type of microbubble used. The contrast medium is eliminated partly through the liver and partly through the pulmonary filter. CEUS contrast agents have few limitations, making them usable even in patients with poor renal function [22].

CEUS has been shown to improve the diagnostic performance of B-mode US [23, 24]. However, its moderate ability to differentiate benign lesions from malignant ones and the lack of consensus on acquisition and interpretation techniques have prevented its routine use [5, 25]. As a result of this, this technique is not included in the BIRADS guidelines [26].

Further studies are needed to determine the potential of CEUS as a diagnostic tool for breast cancer.

### USMI

US molecular imaging (USMI) is an evolving technique that shows promise in the field of oncology. It shares similarities with contrast-enhanced US (CEUS) but has a significant difference in that the biocompatible outer shell of microbubbles has the ability to recognize specific molecular targets, resulting in an in vivo target-ligand bond [21, 27]. This is made possible by the incorporation of specific engineered ligands into the outer shell of the microbubbles, each with a corresponding in vivo target. Upon intravenous injection of the contrast medium, it selectively accumulates in the biological sites where the chosen biological target is present [21].

**Fig. 3:**
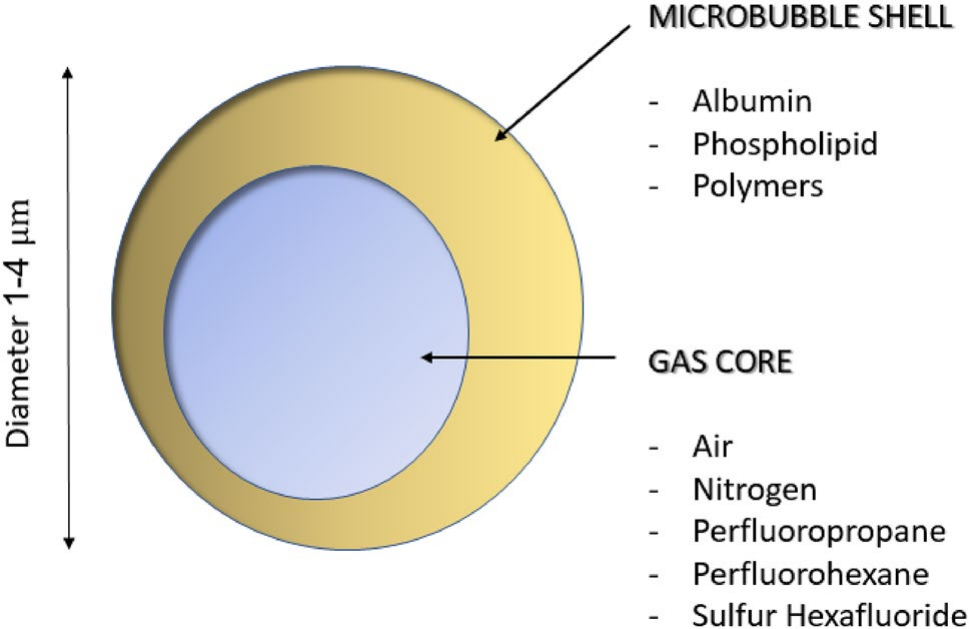
Schematic representation of a microbubble, including examples of various outer shells and inner gas contents

A particular evolution of the CEUS technique is US molecular imaging (USMI), which is emerging as a promising tool in oncology. Even though USMI has several points in common with CEUS, there is a substantial difference between these two techniques: the biocompatible outer shell of USMI is able to recognise a specific target at the molecular level, establishing an in vivo target-ligand bond [21, 27]. All this is made possible by adding to the outer shell of the microbubbles specific engineered ligands, each with a corresponding in vivo target (Fig. 3). Once the contrast medium is injected intravenously, it will tend to accumulate selectively in the biological sites where the chosen biological target is present [21].

The ability of microbubbles to bind to their endogenous target is determined by a balance between the adhesive force and blood flow. When microbubbles enter the bloodstream, they come into contact with specific transmembrane receptors expressed by ECs. If the kinetics of adhesion between the microbubbles and ECs are favorable, the bond occurs. The strength of the receptor-ligand bond and the availability of binding sites determine the degree of retention of the contrast medium. If the adhesive force is greater, the microbubbles will remain adhered to their endogenous receptor. Over time, a fraction of microbubbles adhere to the target with each cardiac cycle, resulting in accumulation [28, 29]. The accumulated microbubbles generate a US signal that is more sensitive and significantly higher than that of the microbubbles that are still freely circulating, utilizing the same image detection technologies used by CEUS without molecular target [29]. Since the background signals from freely circulating microbubbles tend to decrease rapidly, USMI can be performed within a few minutes of intravenous administration of the contrast medium [21].

**Fig. 4:**
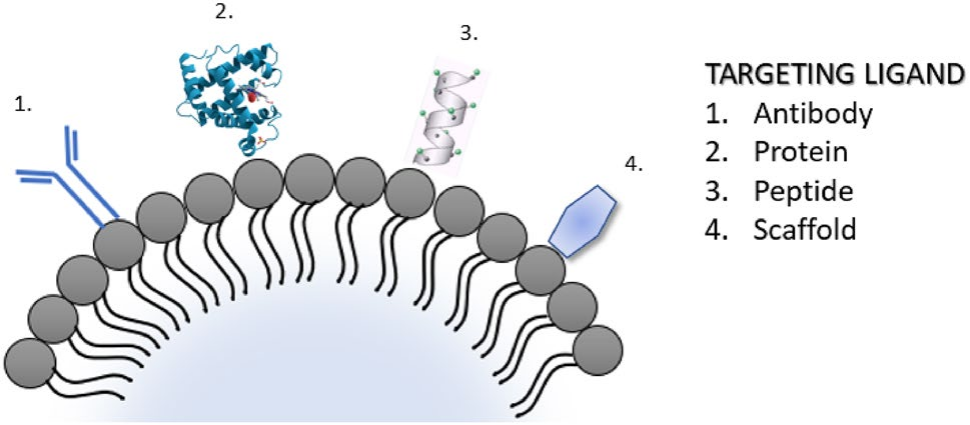
Specific molecular ligands of microbubbles: antibodies, peptides, proteins, natural scaffolds, or engineered scaffolds

The method for producing microbubbles with specific ligands is similar to that of conventional microbubbles, with the addition of an extra step that involves inserting the ligand into the microbubble shell. The specific molecular ligand can be in the form of antibodies, peptides, proteins, natural or engineered scaffolds (Fig. 4), and it can be incorporated during or after the synthesis of microbubbles.

The ligands can be bound to the phospholipid shell in a direct way or through conjugation. The methods of chemical conjugation can exploit covalent bonds, which occur thanks to chemical reactions between the ligand and the chemical groups exposed on the surface of the microbubbles. Alternatively, it can be accomplished with non-covalent methods, through conjugations using biotin/streptavidin (or avidin, alternatively) and/or poly-ethylene–glycol (PEG) chains. In general, however, the use of non-covalent conjugation with biotin-avidin is limited by the risk of immunogenicity, making it unsuitable for the clinical setting [21, 29].

**Fig. 5:**
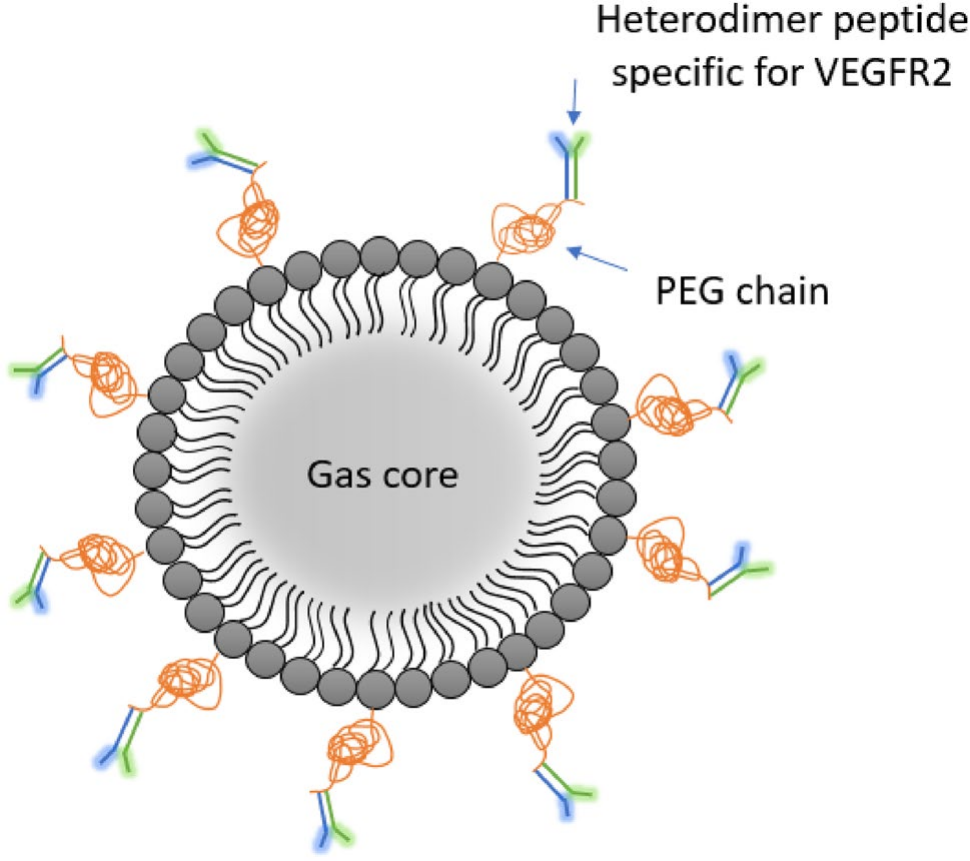
Scheme of a BR55 molecule. The inner gas core is surrounded by a phospholipid shell. The ligand consists of a heterodimeric peptide whose target is represented by the KDR insert of VEGFR-2, bound to the phospholipid shell through a PEGylated chain

Several biomarkers have been investigated as endogenous targets for USMI, of which the most studied are some types of integrin, endoglin and, above all, VEGFR-2. The latter is the molecular target of the first compound that entered the clinical phases of drug development and known as BR55 (Bracco Suisse SA, Geneva, Switzerland). It consists of a gas core (a mixture of perfluorobutane and nitrogen), surrounded by a phospholipid shell with an average diameter of 1.5 µm: the ligand is a heterodimeric peptide with target represented by VEGFR-2, which is also known as KDR (kinase-insert domain-receptor), covalently conjugated through its amino-terminal group with a pegylated lipopeptide construct (Fig. 5) [21]

BR55, the first microbubble contrast agent targeting VEGFR-2, has been shown to be safe in all patients during phase I clinical trials. A few minutes after intravenous injection of BR55, the accumulation of microbubbles is exclusively localized at the site of the malignant lesion, corresponding to an overexpression of VEGFR-2. The intensity of the signal reflects the tumor vascularization and levels of VEGFR-2 produced by the vascular endothelium [30].

The potential of BR55 also goes beyond early tumour diagnosis. Given the central role that VEFR-2 plays in neoangiogenesis, it is the target of numerous anti-tumour therapies and quantitative imaging of its expression is proving to be a new multiparametric approach to breast cancer. In fact, BR55 has been shown to become a tool for predicting and monitoring the response to anti-angiogenic therapy in patients: on the one hand, BR55 can discriminate tumour lesions that are able to respond to therapy with bevacizumab (anti-VEGF monoclonal antibody) and sorafenib (TKI inhibitor of tyrosine kinases, including VEGFR), on the other hand, the method is sensitive to any mutations that the therapy itself induces in the tumour and that may, therefore, emerge during therapeutic treatment [32].

Recently, promising results have also emerged from studies on animals in vivo regarding the research and diagnosis of possible liver metastases from breast cancer, which share with the primary tumour the same altered molecular pathways and for which BR55 has shown some efficacy [31].

In the future, USMI with BR55 may represent a fundamental technique for providing a comprehensive range of information on the tumour, from the initial diagnosis to the evaluation of the response to treatment, assisting clinicians in setting up and modifying the therapeutic protocol for each individual patient [32].

## The future: beyond microbubbles

One of the main limitations of USMI microbubbles is related to their size, which prevents extravasation outside the vascular bed. Therefore, it is not possible to recognize molecular targets in the extravascular setting, such as tumour cells.

**Fig. 6:**
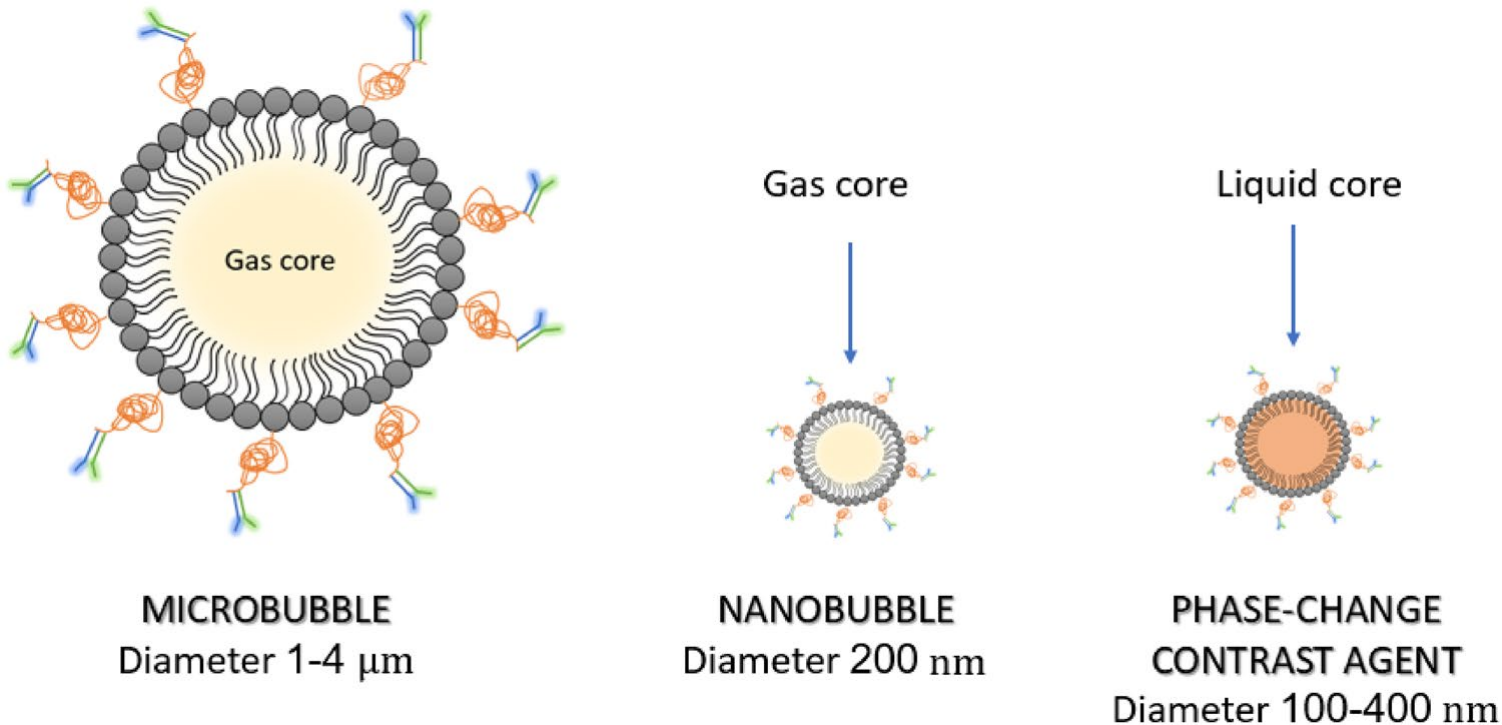
Molecular schematic representation of (from left to right) microbubbles, nanobubbles, and phase-change contrast agents

Nanomedicine has made rapid advances and offers possibilities for new types of contrast media with smaller molecules that have a diameter of less than 1 µm. These smaller molecules, collectively called nanosized US contrast agents (nUCAs), have the potential to overcome this limitation (Fig. 6). nUCAs can be divided into three types: nanobubbles (NBs), phase-change droplets (PCDs), and gas generating nanoparticles (GGNPs) [33].

Nanobubbles (NBs) have a molecular structure similar to that of microbubbles, comprising an inner core consisting of gas enclosed within an outer phospholipid or polymer shell. However, their size allows for accumulation within the tumour interstitial space [34, 35]. As with microbubbles, it is possible to conjugate ligand molecules to the surface of nanobubbles, making them specific for certain tumour targets. However, in vivo, these compounds have displayed low stability that ultimately affected their clinical application [33].

Phase-change contrast agents (PCCAs) are nanoemulsions that can extravasate and accumulate at the site of interest, retaining their morphology and initial size. These agents undergo a transition from the liquid to gaseous state following activation by exposure to a high-energy source, such as US. This results in production of microbubbles in-situ [33].

Lastly, gas-generating nanoparticles incorporate a reactive group in their molecular structure that can produce gas (i.e., oxygen or carbon dioxide) once the pathological site of interest is reached. The initial in vivo experiments have shown considerable gas production at the site of interest, which persists for 4 to 24 h after intravenous injection of the contrast medium [33]. However, none of these contrast media has yet reached the clinical phases of studies [35].

## Conclusion

In the last few decades, breast US has emerged as an essential examination for the diagnosis of breast cancer. Technological advancements, beginning with B-mode evaluation, and progressing through echo-colour-Doppler and CEUS techniques, have led to an expansion of the information obtained from each investigation, thereby allowing for increasingly refined tumour diagnosis.

In modern times, the advent of ultrasonographic molecular imaging (USMI) represents an innovative approach to US diagnostic investigation that is perfectly aligned with personalised medicine. This approach goes beyond mere morphological or functional data and delves deeper into the molecular level to identify the genetic alterations that underpin the oncological transformation of breast tissue. It is now widely accepted that a single tumour lesion results from a heterogeneous group of tumour clones, each with molecular peculiarities that develop under the pressure of biological selection and later resistances to therapies undertaken. The molecular alterations then translate into functional characteristics, such as invasive potential, therapeutic, and biological aggressiveness.

In conclusion, despite being subject to ongoing research and refinement, these agents demonstrate immense potential, with initial findings showing promising results. The successful development of these agents requires close collaboration and coordination among medical, chemical, and biological experts. Therefore, fostering interdisciplinary cooperation is recommended to maximize the progress and application of these agents in various fields.
