# Correction: Exploring the potential of contrast agents in breast cancer echography: current state and future directions

**DOI:** 10.1007/s40477-024-00892-x

**Published:** 2024-05-10

**Authors:** Oriana Monzeglio, Vittoria Maria Melissa, Sara Rodolfi, Eleonora Valentini, Alessandro Carriero

**Affiliations:** 1 Department of Diagnosis and Treatment Services, Radiodiagnostics and Interventional Radiology, AOU Maggiore Della Carità, Corso Mazzini 18, 28100 Novara, Italy; 2 Department of Translation Medicine, University of Eastern Piemonte UPO, Via Solaroli 17, 28100 Novara, Italy

**Correction: Journal of Ultrasound (2023) 26:749–756** 10.1007/s40477-023-00809-0

In page 5, 2nd column, 2nd paragraph has been incorrectly published in the original publication as:

Several biomarkers have been investigated as endogenous targets for USMI, of which the most studied are some types of integrin, endoglin and, above all, VEGFR-2. The latter is the molecular target of the first compound that entered the clinical phases of drug development and approved by the FDA, known commercially as BR55® (Bracco Suisse SA, Geneva, Switzerland). It consists of a gas core (a mixture of perfluorobutane and nitrogen), surrounded by a phospholipid shell with an average diameter of 1.5 μm: the ligand is a heterodimeric peptide with target epresented by the KDR insert of VEGFR-2, covalently conjugated through its amino-terminal group with a pegylated lipopeptide construct (Fig. 5) [21]

The complete correct paragraph should be read as follows.

Several biomarkers have been investigated as endogenous targets for USMI, of which the most studied are some types of integrin, endoglin and, above all, VEGFR-2. The latter is the molecular target of the first compound that entered the clinical phases of drug development and known as BR55 (Bracco Suisse SA, Geneva, Switzerland). It consists of a gas core (a mixture of perfluorobutane and nitrogen), surrounded by a phospholipid shell with an average diameter of 1.5 μm: the ligand is a heterodimeric peptide with target represented by VEGFR-2, which is also known as KDR (kinase-insert domain- receptor), covalently conjugated through its amino-terminal group with a pegylated lipopeptide construct (Fig. 5) [21]

The original article has been corrected.

